# Triple-Negativity Identifies a Subgroup of Patients with Better Overall Survival in Essential Thrombocythemia

**DOI:** 10.3390/hematolrep14030037

**Published:** 2022-08-24

**Authors:** Marco Santoro, Vincenzo Accurso, Salvatrice Mancuso, Mariasanta Napolitano, Marta Mattana, Giorgia Vajana, Federica Russello, Sergio Siragusa

**Affiliations:** 1 Department of Health Promotion, Mother and Child Care, Internal Medicine and Medical Specialties (PROMISE), Hematology Unit, University of Palermo, Via del vespro 129, 90127 Palermo, Italy; 2 Hematology Division, University Hospital Policlinico “Paolo Giaccone”, Via del vespro 129, 90127 Palermo, Italy

**Keywords:** essential thrombocythemia, triple-negative, triple-negativity, survival

## Abstract

Essential thrombocythemia, as defined by the WHO in 2016, is a Philadelphia-negative chronic myeloproliferative neoplasm showing a better prognosis than polycythemia vera and myelofibrosis. In a variable percentage, patients with essential thrombocythemia show none of the known driver-gene mutations that may occur on JAK2, CALR, and MPL genes. Such patients are classified as triple-negative and their clinical features and prognosis have not been described with precision yet. In this study, we evaluated some of the characteristics of this population by comparing them with those of patients with driver-gene mutated ET. Data from 266 consecutive essential thrombocythemia patients were analysed. Triple-negative patients had a significantly lower symptom load and a lower frequency of splenomegaly at diagnosis. The results show that the rate of thrombosis was equal in the two subgroups. Overall survival was slightly better in the triple-negative group of patients.

## 1. Introduction

Philadelphia-negative chronic myeloproliferative neoplasms (MPNs) were initially identified in 1951 as myeloproliferative disorders [1], although only in 2000 were these diseases definitely classified as malignant neoplasms, according to the 3rd edition of the International Classification of Diseases for Oncology [2]. The V617F mutation of the JAK2 gene was discovered in 2005 [3], and subsequently mutations of the thrombopoetin receptor gene (MPL) [4,5]; mutations of the JAK2 exon-12 [6]; and, finally, mutations of the calreticulin gene (CALR) [6] were demonstrated to be associated with MPN pathogenesis in 2006, 2007, and 2013, respectively. These recent discoveries are important contributions to knowledge of the MPNs’ molecular mechanisms. In 2008, the World Health Organization (WHO) redefined the myeloproliferative disorders as myeloproliferative neoplasms [7] and in 2016 made a further revision of the case definition for Essential Thrombocythemia (ET), Polycythemia Vera, and Primary Myelofibrosis, introducing the distinction between pre-fibrotic and overt Myelofibrosis [8].

Among the three major myeloproliferative diseases classified by the WHO, ET is more indolent with a 5-year survival rate of 85–91%; it occurs frequently in the female sex [9,10], with a median age at diagnosis of 67 years in the USA. ET is classified as a rare disease, with an estimated incidence of 1.1–2.0 cases per 100,000 persons per year and a prevalence of 24–58 per 100,000 persons, in 2016 [11]. Some authors have shown a significant reduction in age at diagnosis [12,13]. Clinical manifestations in essential thrombocythemia are similar to those seen in other myeloproliferative diseases and sometimes can be particularly severe. In 2006, Mesa et al. have conducted a survey on 1179 patients with MPN and showed that 70% of them report constitutional symptoms and splenomegaly-related symptoms. The most frequent symptom is various-grade asthenia, reported by 81% of the patients [14]. 

However, the main features of ET are the marked thrombocytosis on the blood count and the increased thrombotic risk. In fact, amongst 1297 patients with ET, 17.8% have reported a thrombus prior to or at the time of ET diagnosis [15]. Thrombotic risk is currently estimated by applying the so-called IPSET-t score, which divides the distinction into four risk categories: very low, low, intermediate, and high risk [16]. Acetylsalicylic acid alone is provided for patients in very-low-risk or low-risk, while cytoreductive therapy (hydroxyurea, busulfan, or interferon) is indicated for high-risk and to be taken into consideration—without being mandatory—for intermediate-risk patients [17]. 

With regards to mutational status, the V617F JAK2, CALR, and MPL mutations (driver mutations) are harbored by the pathological-cell population in 50–60%, 15–30%, and 1–5% of the ET cases, respectively. In the remaining percentage of patients with ET, none of the driver mutations can be found, and these patients are thus classified as “triple-negative” (TN). In the past, some authors have highlighted peculiar characteristics in this population [18]. Starting from these evidences, we aimed to describe the clinical characteristics of the TN-ET patients followed at our centre and to determine whether they have a better prognosis than driver-mutated patients.

## 2. Patients and Methods

We conducted a retrospective analysis on 266 consecutive ET patients referred to our centre from January 1997 to January 2021. As per the definition of triple negativity, in the absence of data for non-driver mutations that may define myeloid clonality, the diagnosis of all the included cases of TN-ET required the exclusion of secondary causes of thrombocytosis as a minor criterion; this diagnostic process followed the 2016 WHO diagnostic criteria for myeloid neoplasms [7]. The median follow-up time is 51.5 months. For each of these patients the absence of the three driver-gene mutations has been determined. 

At diagnosis, MPN-10 score [19] was calculated to evaluate the symptom load, the presence of splenomegaly, the frequency of venous and arterial thromboses or other cardiovascular events, and the eventual evolution into myelofibrosis and/or leukemia. The number of deaths and survival data of these patients were recorded, and the characteristics in triple negative patients were compared to patients with known mutations on the driver genes. A comparison between frequencies was carried out with the chi-square method, a comparison between medians with the Kruskal-Wallis test. Survival rates were calculated with the Kaplan and Meyer method, and the comparison between survival data was carried out with the log-rank test.

## 3. Results

Of the 266 patients included in the study, 192 harboured the V617F mutation of the JAK2 gene (71.8%), 26 patients carried a pathogenic mutation of CALR (9.77%), and 4 patients were mutated on MPL (1.50%). On the other hand, 45 patients showed none of the above-mentioned mutations on the three MPNs driver genes and were thus considered triple-negative MPNs (TN-MPN) (16.92%). 

In the examined population, the median age at diagnosis for the whole ET patients group was 60 years. TN-MPN patients had a median age of 52.3 years (range 14.3–89.7), while driver-mutated patients were significantly older, with a median age of 66.6 years (range 18–90.7) (*p* = 0.00028). Groups of patients according to gender showed that there was a slight prevalence of the female sex (34/45, 75.7%), while driver-mutated patients were female in 146/221 cases (60.1%). This difference does not achieve statistical significance (*p* = 0.21), but remains interesting as a trend. 

Data from MPN-10 score at diagnosis are available for 148 patients, of whom 116 had driver mutations and 32 TN-MPN. The median score is 19 points (range 8–23) in patients with mutations and 13 in TN-MPN patients (range 4–19) (*p* = 0.001). The frequencies of thrombotic and cardiovascular events appear to be similar in the two groups of patients with 12/45 episodes in TN (26.66%) and 59/221 (26.70%) in patients with a mutation. At diagnosis, splenomegaly is more frequently described in TN-MPNs (15.8 versus 6.7% in driver-mutated patients) (*p* = 0.05). 

Evolution into myelofibrosis was diagnosed in 5 patients in the observation period, with further evolution into acute leukemia in 2 cases. Of those, only one case of MF evolution occurred in the TN group. Overall, 28 deaths occurred in patients with mutations on the driver genes (12.7%), while only 3 deaths occurred in TN patients (6.7%) (*p* = 0.2). Results of our study are reported in Table 1 for better visualization (see).

**Figure 1:**
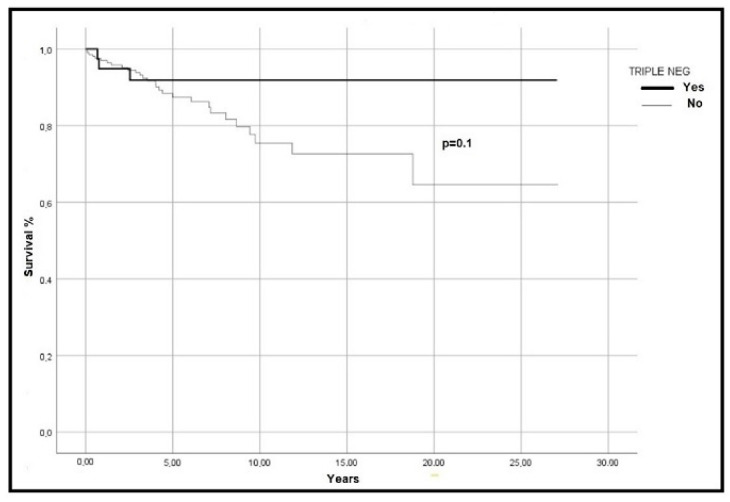
Overall survival in 266 ET patients. The difference between the TN and the driver-gene mutated patients is significant with a *p*-value of 0.1.

Survival curves are slightly more favourable for the TN-MPN group than driver-mutated (*p* = 0.1), as shown in Figure 1. 

## 4. Discussion and Conclusions

The existing literature reports that the expected survival of ET patients reaches about 20 years. A recent review on MPN epidemiology report a median OS of 13–23 years and a 5-year relative survival of 86–91%, with a median age at diagnosis of 67 years [20].

In our study, TN-ET patients show a median survival of 24.5 years, higher than that found in patients with mutations affecting one of the three driver genes (21.7 years). Our finding confirms previous studies that have already reported a slightly better survival for TN ET patients [18,21]. One of the possible reasons that can explain this better outcome is the lower age at diagnosis in TN cases.

Patients with TN ET show a significantly lower symptom load, when it is evaluated by the MPN-10 score, along with a lower frequency of splenomegaly at diagnosis. Splenomegaly is present in about 15% of patients with ET and appears to correlate with a worse prognosis [22]. 

Thromboses occurred without significant differences in triple negative and in driver-gene-mutated patients in our study, in contrast with the existing literature [21]. 

A limitation of our study is the lack of information regarding mutations in other genes that have been associated with myeloid neoplasms. The existing literature does not strongly report the association between the presence of non-driver mutations hitting genes like TET2, SH2B3, and ASXL1 as well as thrombotic events or survival. Moreover, some atypical JAK2, CALR, and MPL mutations have been identified in some TN patients [23,24]. 

Currently, the clinical and molecular characteristics of patients with TN-ET are not well characterized in the existing literature. Overall, our findings, even within the limits of a mono-institutional study, suggest a better prognosis in patients with TN-ET. 

In our single-institution study, the survival data, while not statistically significant, hint at a better prognosis, in terms of survival, in patients with TN-ET.

## Figures and Tables

**Table 1:** Main results of the study described for all ET patients, driver-mutated and triple-negative subgroups. #, absolute number of cases; %, percentage. * MPN10-TSS: Myeloproliferative Neoplasms 10—Total Symptom Score. For the MPN10 item, data were available for 148 patients (116 driver-mut and 32 triple-neg).

	*All* # (%)	*Driver-Mut* #(%)	*Triple-Neg* # (%)	*p*
**Patients**	266 (100)	221 (83.1)	45 (16.9)	-
**Female sex**	180 (63.9)	146 (60.1)	34 (75.7)	0.21
**Median age, years (range)**	60 (14.3–90.7)	66.6 (18–90.7)	52.3 (14.3–89.7)	**0.00028**
Median MPN10-TSS *, score (range)	17 (4–23)	19 (8–23)	13 (4–19)	**0.001**
**Thrombosis**	71 (26.69)	59 (26.7)	12 (26.66)	0.52
**Splenomegaly**	21 (7.8)	14 (6.7)	7 (15.8)	**0.05**
**MF evolution**	5 (1.8)	4 (1.8)	1 (2.2)	0.45
**Death all cases**	31 (11.6)	28 (12.7)	3 (6.7)	0.2

## Data Availability

The data used to support the findings of this study are available from the corresponding author upon request.
